# Acute Phase Reactions After Intravenous Infusion of Zoledronic Acid in Japanese Patients with Osteoporosis: Sub-analyses of the Phase III ZONE Study

**DOI:** 10.1007/s00223-021-00884-7

**Published:** 2021-07-10

**Authors:** Masataka Shiraki, Tatsuhiko Kuroda, Yasuhiro Takeuchi, Toshitsugu Sugimoto, Satoshi Tanaka, Hiroaki Suzuki, Kazuki Hiraishi, Toshitaka Nakamura

**Affiliations:** 1 Department of Internal Medicine, Research Institute and Practice for Involutional Diseases, 1610-1 Meisei, Misato, Azumino, Nagano 399-8101 Japan; 2 Healthcare R&D Center, Asahi Kasei Corporation, 1-1-2 Yurakucho, Chiyoda-ku, Tokyo, 100-0006 Japan; 3 Toranomon Hospital Endocrine Center, 2-2-2 Toranomon, Minato-ku, Tokyo, 105-8470 Japan; 4 Okinaka Memorial Institute for Medical Research, 2-2-2 Toranomon, Minato-ku, Tokyo, 105-8470 Japan; 5 Internal Medicine 1, Shimane University Faculty of Medicine, 89-1 Enya-cho, Izumo, Shimane 693-8501 Japan; 6 Medical Affairs Department, Asahi Kasei Pharma Corporation, 1-1-2 Yurakucho, Chiyoda-ku, Tokyo, 100-0006 Japan; 7 Touto Sangenjaya Rehabilitation Hospital, 1-24-3 Sangenjaya Setagaya-ku, Tokyo, 154-0024 Japan

**Keywords:** Acute phase reaction, Bone mineral density, Bone turnover marker, Osteoporosis, Zoledronic acid

## Abstract

In a clinical trial involving Japanese patients with osteoporosis, post hoc analyses were performed to evaluate the incidence of acute phase reactions (APRs) after infusion of zoledronic acid (ZOL). The results highlighted differences in baseline factors between patients with vs without APRs. Changes in efficacy indicators such as bone turnover markers (BTMs) also showed significant differences. We, therefore, investigated the factors involved in the development of APRs in Japanese patients treated with a once-yearly intravenous infusion of ZOL 5 mg for 2 years by assessing the relation between APRs and efficacy. APRs reported in patients with primary osteoporosis from the ZONE study were analyzed post hoc. Baseline factors were compared in patients with vs without APRs, and changes in BTMs and bone mineral density (BMD) were also investigated. In the ZOL group, 51.2% (169/330) of patients developed APRs after the first infusion and 12.3% (33/268) after the second infusion. Comparison of baseline factors showed that patients without APRs in the ZOL group had a significantly higher neutrophil/lymphocyte ratio, lower serum levels of procollagen type I N-terminal propeptide, older age, and higher likelihood of prior bisphosphonate use vs patients with APRs. Patients with APRs showed significantly higher increases in total hip BMD at 6 and 12 months and larger reductions in BTMs vs patients without APRs. Patient profiles differed significantly between patients with vs without APRs, with APRs after the first infusion of ZOL being related to increases in total hip BMD and suppression of BTMs.

This study is registered with ClinicalTrials.gov (identifier: NCT01522521; January 31, 2012).

## Introduction

Various types of drugs for osteoporosis have been available in the Japanese market, and bisphosphonates are used as a first-line treatment for patients with osteoporosis [1]. However, poor adherence to osteoporosis drugs has been a major concern, with 45.2% of patients being noncompliant within 1 year after initiation of treatment and 52.1% discontinuing treatment within 5 years [2]. The most common reason for osteoporosis treatment discontinuation is drug-induced adverse events (AEs), followed by lack of awareness. Among the AEs associated with bisphosphonates, upper gastrointestinal (GI) tract distress is the most prominent reason for discontinuing oral bisphosphonates [3].

Zoledronic acid (ZOL) is a potent bisphosphonate that is administered as a 5-mg intravenous infusion once yearly [4–9]. In a previous clinical study—the Health Outcomes and Reduced Incidence with Zoledronic acid Once Yearly-Pivotal Fracture Trial (HORIZON-PFT)—treatment with ZOL for up to 3 years showed a significant and sustained decrease in the risk of vertebral, hip, and other osteoporotic fractures and was well tolerated in patients with osteoporosis [4]. Therefore, parenteral administration of ZOL is expected to be a promising therapeutic option for osteoporosis to avoid GI distress, resulting in better adherence rates [10–13].

The phase III ZOledroNate treatment in Efficacy to osteoporosis (ZONE) study demonstrated the efficacy and safety of ZOL in Japanese patients with primary osteoporosis [14]. Patients in the ZOL group had significantly lower incidences of vertebral fractures than those in the placebo group, with a 65.8% relative risk reduction. ZOL also significantly decreased the cumulative incidence of nonvertebral fractures.

However, ZOL has been reported to be associated with acute phase reactions (APRs) such as pyrexia and influenza-like symptoms [15, 16]. Since AEs involving APRs are considered a major cause of reduced adherence rates, strategies to prevent and/or to better manage APRs are warranted.

Antecedent reports have identified the factors associated with APRs, and these parameters may help predict the development of APRs [17, 18]. Adequate levels of serum 25-hydroxy vitamin D (25(OH)D) and hydration before ZOL infusion have been reported to lower the incidence of APRs [19–21]. Furthermore, patient ethnicity, especially Asian ethnicity, has been reported to increase the incidence of APRs, whereas prior use of bisphosphonates has been reported to reduce the incidence of APRs [15]. However, the study by Reid et al. [15] did not specifically include Japanese subjects; therefore, whether or not these factors are generalizable to Japanese patients with osteoporosis as well remains unknown.

These sub-analyses of data from the ZONE study were conducted to (1) investigate the patient baseline factors associated with the incidence rates of APRs in Japanese patients and (2) investigate whether the effects of ZOL treatment are related to the occurrence of APRs. Potential measures to prevent APRs and enhance their management upon occurrence were further considered.

## Patients and Methods

### Study Design and Population

This was a post hoc analysis of APRs after treatment with intravenous ZOL in a multicenter, randomized, double-blind, placebo-controlled, parallel-group comparative clinical study in Japanese patients with primary osteoporosis. The study design and population have been described previously [14]. Male and female patients aged ≥ 65 and ≤ 89 years with a diagnosis of primary osteoporosis and a vertebral fracture at the time of screening were included in the study. Patients diagnosed with secondary osteoporosis, those with a concomitant uncontrolled medical disorder, or those receiving bisphosphonate treatment within 2 years before the start of the study were excluded.

The study was conducted according to the ethical principles of the Declaration of Helsinki, the International Conference on Harmonisation Good Clinical Practice guidelines, and other applicable laws and regulations. The protocol was reviewed and approved by the institutional review boards of the participating study sites. All patients provided written informed consent before the initiation of the study. The present study is registered with ClinicalTrials.gov (identifier: NCT01522521).

### Patient Disposition

Overall, 665 patients were randomized to receive ZOL (*n* = 333) or placebo (*n* = 332), of whom 542 (ZOL, *n* = 258; placebo, *n* = 284) completed the study.

The present analysis set included 330 patients from the ZOL group and 331 patients from the placebo group who fulfilled the criteria for the full analysis set.

### Treatments

Patients received either a once-yearly intravenous infusion of ZOL (5 mg ZOL in 100 mL) or placebo over 15 min at baseline and at 12 months and were monitored for 2 years (104 weeks). Patients also received once-daily supplemental therapy with calcium 610 mg, vitamin D_3_ 400 IU, and magnesium 30 mg after dinner, starting from the day of providing written informed consent until the end of the study period.

### Data Collection

Baseline factors including age, body mass index (BMI), postmenopausal duration (in women), recent alcohol consumption, smoking status, and prior bisphosphonate use were assessed. Baseline blood samples were used to measure the differential leukocyte count, neutrophils, lymphocytes, monocytes, eosinophils, and basophils. The neutrophil/lymphocyte ratio (NLR) was also calculated, and corrected serum levels of calcium, phosphorus, and 25(OH)D were measured. Data for bone mineral density (BMD) by dual X-ray absorptiometry of the lumbar spine (L2-4), femoral neck, and total hip at baseline; bone resorption markers (serum C-terminal telopeptide of type I collagen degradation products [CTx] and tartrate-resistant acid phosphatase 5b [TRACP-5b]); and bone formation markers (serum bone-specific alkaline phosphatase [BAP] and procollagen type I N-terminal propeptide [P1NP]) were measured and further analyzed. The methods used for the measurement of BMD and bone turnover markers (BTMs) have been illustrated in the ZONE study [14].

### Acute Phase Reactions

AEs were monitored from the day of the first infusion to the end of the study period (104 weeks after the first infusion or at the time of study discontinuation). All AEs were coded using the Japanese version of the Medical Dictionary for Regulatory Activities (MedDRA/J) version 17.1. The name, system organ class/preferred term, severity, time of onset (1–3 days after the infusion), and causal relationship of the AE with the study drug were analyzed for all AEs by treatment group. For this analysis, the occurrence of one or more of the following AEs within 3 days after infusion, as described in the previous report [15], was regarded as an APR: pyrexia, arthralgia, influenza-like illness, myalgia, headache, malaise, and chills.

### Statistical Analysis

Quantitative variables are reported as mean ± standard deviation (SD). Categorical variables are reported as numbers and percentages. Patients were categorized into 2 groups: patients who developed APRs (APR+ group) and patients who did not develop APRs (APR− group). To identify the risk factors for APRs after treatment with ZOL, baseline factors were compared between the APR+ and APR− groups using an analysis of variance (ANOVA) or the chi-square test. Factors that showed a significant association with APRs were analyzed using a multiple regression analysis to assess their contribution to the incidence of APRs. A *p* value of < 0.05 was considered statistically significant in all tests.

## Results

### Patient Demographics and Baseline Factors

Patient demographics and baseline factors of the analysis set were identical to those reported in the published ZONE study [7]. The mean ± SD age of patients in the ZOL and placebo groups was 74.0 ± 5.4 and 74.3 ± 5.4 years, respectively, with 309 and 312 women in the ZOL and placebo groups, respectively. No remarkable differences in other factors were observed between the groups. The duration of infusion for both the first and second infusions was ≥ 15 min in all patients. The mean duration of infusion in the ZOL group was 27.5 min and 27.8 min for the first and second infusions, respectively.

### Acute Phase Reactions

Table 1 summarizes the AEs identified as APRs.

**Table 1:** Incidence of APRs

AEs defined as APRs *n* (%)	First infusion			Second infusion		
	ZOL *n* = 330	Placebo *n* = 331	*p* value	ZOL *n* = 268	Placebo *n* = 287	*p* value
Total APRs	169 (51.2%)	11 (3.3%)	< 0.001	33 (12.3%)	7 (2.4%)	< 0.001
Pyrexia	128	5		20	3	
Arthralgia	33	1		1	1	
Influenza-like illness	23	0		2	0	
Myalgia	24	0		3	0	
Headache	18	1		4	1	
Malaise	19	5		9	2	
Chills	15	1		3	0	

*AE* adverse event, *APR* acute phase reaction, *ZOL* zoledronic acid

In the ZOL group, 51.2% (169/330) of patients developed APRs after the first infusion and 12.3% (33/268) after the second infusion (Table 1). The incidences of APRs in the placebo group after the first and second infusions were 3.3% and 2.4%, respectively, which were significantly (*p* < 0.001) lower than those in the ZOL group. Overall, 32 patients (9.7%) experienced APRs after both the first and second infusions in the ZOL group, compared with no patients in the placebo group.

In both groups, most of the APRs as judged by the investigators were mild, with no severe APRs. The classification did not take into account whether a patient had received rescue medication or not. The time to resolution of APRs ranged from 1 to 3 days after onset. Pyrexia was the most frequent APR after both the first and second infusions of ZOL (38.8% [128/330] and 7.5% [20/268], respectively). No patients with pyrexia after the first infusion had worsening of pyrexia after the second infusion.

### Background Factors of Patients with APRs

Baseline patient factors were compared between patients with and those without APRs after the first infusion of ZOL. Furthermore, we investigated the association of sex with the development of APRs. The results showed that the mean age was lower in the APR+ group vs the APR− group (*p* = 0.031). More patients in the APR− group had received prior bisphosphonates (*p* = 0.016; Table 2). Significant differences between the APR+ and APR− groups were also noted with regard to the neutrophil count (*p* < 0.001) and lymphocyte count (*p* < 0.001) in the differential leukocyte count, with a significantly lower NLR in the APR+ group. CTx and P1NP were significantly higher in the APR+ group (*p* = 0.011 and *p* = 0.001, respectively). No significant differences were observed in serum 25(OH)D levels at baseline in both APR groups. Furthermore, we compared the incidence of APRs by 25(OH)D status (deficiency: < 20 ng/mL, insufficiency: 20–30 ng/mL, and sufficiency: ≥ 30 ng/mL) at baseline. However, no statistical differences were observed (*p* = 0.550 in the ZOL group). Since 25(OH)D levels at the first infusion did not alter the expression of APRs, we considered that analysis after the second infusion was not necessary.

**Table 2:** Baseline factors of patients in the ZOL group by development of APRs

Variables	APR+ *n* = 169	APR− *n* = 161	*p* value
Age (years)	73.3 ± 5.1	74.6 ± 5.5	0.031
Sex			
Female	162	147	0.088
Male	7	14	
Years since menopause^a^	23.0 ± 6.0	24.0 ± 6.7	0.174
BMI (kg/m)^2^	23.5 ± 3.4	23.2 ± 3.1	0.439
25(OH)D (ng/mL)	26.3 ± 6.7	26.0 ± 6.4	0.729
Corrected calcium (mg/dL)	9.3 ± 0.3	9.3 ± 0.3	0.354
Phosphate (mg/dL)	3.7 ± 0.4	3.6 ± 0.4	0.454
BMD^b^ (g/cm^2^)			
Lumbar	0.683 ± 0.100	0.677 ± 0.089	0.723
Femoral neck	0.525 ± 0.079	0.529 ± 0.081	0.693
Total hip	0.652 ± 0.095	0.649 ± 0.100	0.820
Creatinine clearance (mL/min)			
< 40	27	23	0.912
40–60	113	110	
≥ 60	29	28	
Smoking			
Yes	8	10	0.555
No	161	151	
Alcohol consumption			
Yes	4	3	0.751
No	165	158	
Diabetes mellitus			
Yes	12	14	0.591
No	157	147	
Back pain			
Yes	21	16	0.473
No	148	145	
Prior bisphosphonate use			
Never used	159	139	0.016
Used, but 2 years or longer washout	10	22	
Prior calcitonin use			
Used with washout	5	12	0.062
Never used	164	149	
Prior NSAID use			
Yes	47	58	0.109
No	122	103	
Leukocytes (count, × 10^3^/μL)	5.1 ± 1.3	5.3 ± 1.6	0.164
Neutrophils (count, × 10^3^/μL)^c^	2.9 ± 1.3	3.2 ± 1.0	< 0.001
Lymphocytes (count, × 10^3^/μL)^c^	1.7 ± 0.4	1.6 ± 0.5	< 0.001
Monocytes (count, × 10^3^/μL)^c^	0.35 ± 0.09	0.36 ± 0.11	0.890
Eosinophils (count, × 10^3^/μL)^c^	0.13 ± 0.03	0.14 ± 0.04	0.611
Basophils (count, × 10^3^/μL)^c^	0.02 ± 0.01	0.02 ± 0.01	0.953
CTx (ng/mL)	0.43 ± 0.19	0.38 ± 0.17	0.011
TRACP-5b (mU/dL)	425.2 ± 161.7	407 ± 132.6	0.268
BAP (µg/L)	17.5 ± 6.5	16.4 ± 6.5	0.127
P1NP (ng/mL)	50.8 ± 22.0	43.4 ± 18.8	0.001

Values are presented as number (*n*) or mean ± SD

*APR* acute phase reaction, *BAP* serum bone-specific alkaline phosphatase, *BMD* bone mineral density, *BMI* body mass index, *CTx* serum C-terminal telopeptide of type I collagen degradation products, *NSAID* nonsteroidal anti-inflammatory drug, *P1NP* procollagen type I N-terminal propeptide, *SD* standard deviation, *TRACP-5b* tartrate-resistant acid phosphatase 5b, *ZOL* zoledronic acid, *25(OH)D* 25-hydroxy vitamin D

^a^Available for 162 and 147 patients in the APR+ and APR− groups, respectively

^b^Lumbar BMD values available for 79 and 70 patients and femoral neck and total hip BMD values available for 122 and 108 patients in the APR+ and APR− groups, respectively

^c^Values available for a total of 168 patients in the APR+ group

We performed a stepwise multivariate regression analysis using the background factors that had a significant association with APRs as variables (age, P1NP, NLR, and prior bisphosphonate use). Age (*p* = 0.050), NLR (*p* = 0.001), and P1NP (*p* = 0.002) were statistically significantly associated with the incidence of APRs, whereas prior bisphosphonate use was not (*p* = 0.082; Table 3).

**Table 3:** Multivariate logistic regression analysis for APRs

Variables	Estimate	SE	*p* value
Increasing age	− 0.010	0.005	0.050
Increasing NLR levels	− 0.062	0.018	0.001
Increasing P1NP levels	0.004	0.001	0.002
Prior bisphosphonate use, yes vs no	− 0.157	0.090	0.082

*APR* acute phase reaction, *NLR* neutrophil/lymphocyte ratio, *P1NP* procollagen type I N-terminal propeptide, *SE* standard error

### Comparison of Efficacy Indicators of ZOL in Patients with and Those Without APRs

**Fig. 1:**
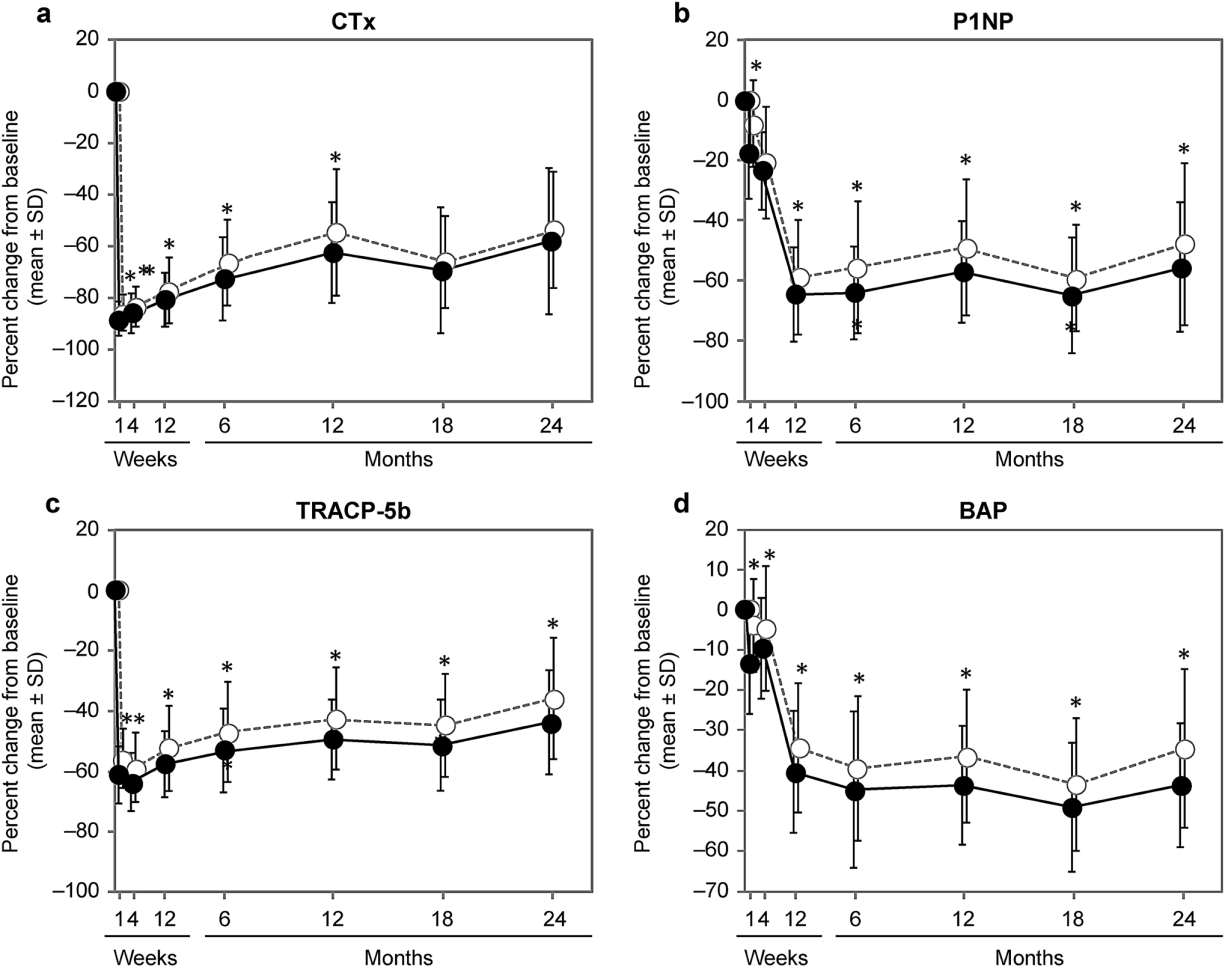
Percent changes from baseline in BTMs with or without APRs. **a** CTx, **b** P1NP, **c** TRACP-5b, and **d** BAP. *p < 0.05 between groups; values are mean ± SD. Solid line, closed circles: patients with APRs; dotted line, open circles: patients without APRs. *APR* acute phase reaction, *BAP* serum bone-specific alkaline phosphatase, *BTM* bone turnover marker, *CTx* serum C-terminal telopeptide of type I collagen degradation products, *P1NP* procollagen type I N-terminal propeptide, *SD* standard deviation, *TRACP-5b* tartrate-resistant acid phosphatase 5b

Patients who experienced APRs after the first infusion of ZOL showed a greater decrease from baseline in the levels of CTx, P1NP, BAP, and TRACP-5b at almost all measurement time points (Fig. 1).

**Fig. 2:**
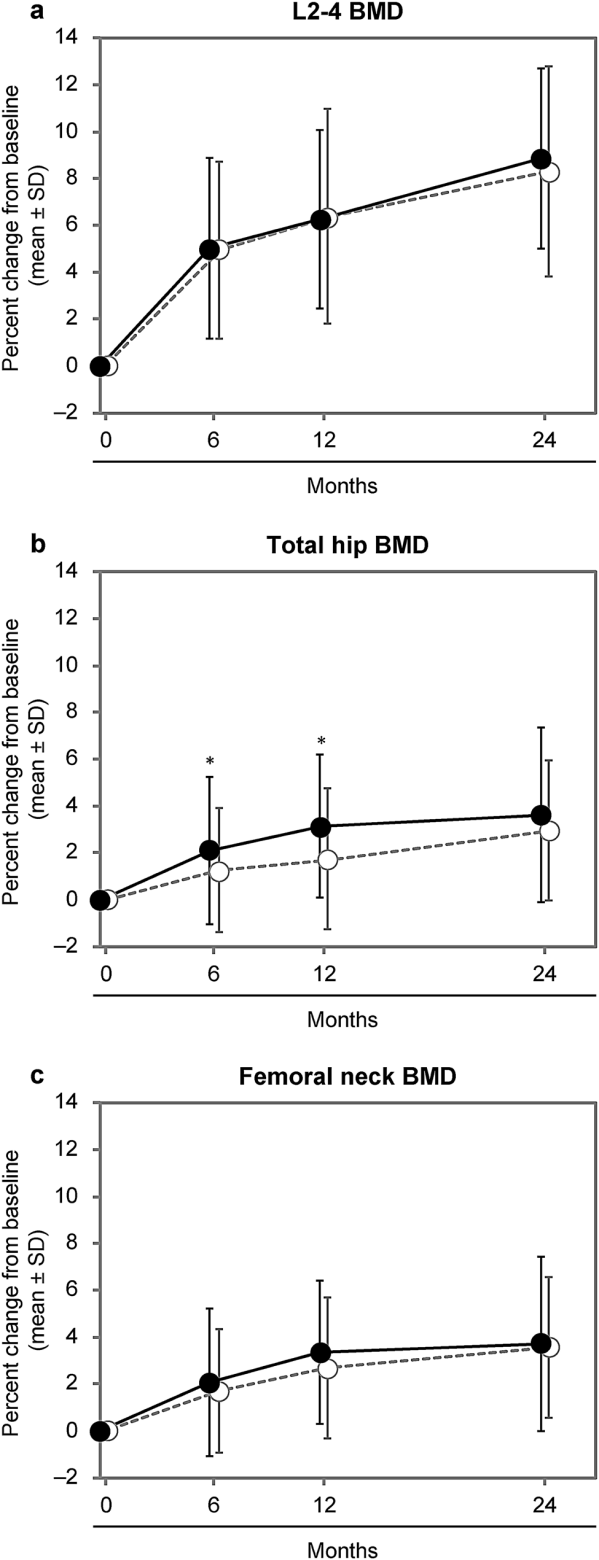
Percent changes from baseline in BMD with or without APRs. **a** lumbar 2–4, **b** total hip, and **c** femoral neck BMD. Solid line, closed circles: patients with APRs; dotted line, open circles: patients without APRs. *p < 0.05 between groups; values are mean ± SD. *APR* acute phase reaction, *BMD* bone mineral density, *SD* standard deviation

In terms of BMD, the APR+ group showed a higher increase in the total hip BMD from baseline at 6 and 12 months compared with the APR− group, but not thereafter. In contrast to the total hip BMD, no difference was observed for the L2–4 BMD and femoral neck BMD (Fig. 2).

## Discussion

In the present analysis, we assessed the incidence of APRs with 2 consecutive once-yearly infusions of ZOL in Japanese patients with primary osteoporosis. We identified 7 AEs that were considered as APRs, namely, pyrexia, arthralgia, influenza-like symptoms, myalgia, headache, malaise, and chills. As reported in previous studies conducted overseas, the APRs observed in Japanese patients were mild in severity and transient, and fewer patients had APRs after the second infusion of ZOL than after the first infusion. Among patients with pyrexia in the ZOL group, the body temperature increased up to 24 h and then gradually decreased to the normal temperature over 72 h, which is consistent with previous reports [17, 18]. Overall, the incidence rates and factors of APRs reported in the present study were similar to those reported in the previous HORIZON study [4, 14].

In this study, the baseline factors significantly related to APRs were age, prior bisphosphonate use, serum levels of CTx and P1NP, and NLR. A stepwise multivariate regression analysis of the prognostic factors of APRs showed that low NLR, high P1NP, and patient age were significant, but prior bisphosphonate use was not. This may be due to the small number of patients with prior bisphosphonate use and because of the 2-year washout period before patients received the infusion of ZOL. No significant differences were found in several other factors, including the incidence rate of APRs by baseline serum 25(OH)D levels in the present study, although previous studies have shown significant differences [19, 20]. This discrepancy in 25(OH)D may be explained by the differences in the levels of serum 25(OH)D before treatment. The mean 25(OH)D level in the APR− group in this study was relatively lower (26.0 ng/mL) compared with that in patients without APRs in a previous report (47.2 ng/mL) [19]. A higher level that was closer to the normal level of 25(OH)D may have decreased the incidence of APRs. In clinical practice, supplementation with native vitamin D should be considered for patients with 25(OH)D insufficiency.

Inhibition of farnesyl pyrophosphate synthase (FPPS) has been reported to have an association with the development of APRs. Increasing the production of isopentenyl pyrophosphate (IPP) by inhibiting FFPS stimulates the immunological system, leading to the release of tumor necrosis factor (TNF)-alpha, interleukin (IL)-6, or interferon (IFN)-γ from peripheral blood γδ T cells [22–26]. In our study, significantly higher levels of lymphocytes and lower levels of neutrophils were observed at baseline in the ARP+ group, which is consistent with previous reports on APRs related to ZOL [27]. Although we did not measure the percentage of γδ T cells in lymphocytes, based on previous reports, it is assumable that APR+ patients had a higher percentage of γδ T cells [20, 24, 26].

The levels of CTx and P1NP at baseline were higher in the APR+ group compared with that in the APR− group. Kim et al. have reported that urinary N-terminal telopeptide of type I collagen (NTx) and BAP were correlated with the level of serum high-sensitivity C-reactive protein (CRP) in healthy pre- and postmenopausal Korean women [28]. Furthermore, Sykiotis et al. reported that the mean level of urine NTx at baseline was higher in patients (*n* = 26) who experienced APRs after the first infusion of ZOL compared with the baseline NTx at the second infusion of ZOL in the same patients [29]. In their study, only 2 of the 26 patients experienced mild APRs after the second infusion of ZOL. They also reported that the degree of urine NTx decline from baseline was correlated with CRP. Based on these reports, it is possible that patients with higher BTMs might have had a low-grade systemic inflammatory status, and, therefore, APRs might have been easily evoked in such patients.

The aforementioned findings therefore suggest that patients with higher bone turnover may release more inflammatory cytokines after infusion of ZOL due to the increased production of IPP. We also observed that patients in the APR+ group had greater inhibition of CTx and P1NP after infusion of ZOL, which could have had a role in the higher increase in total hip BMD at 6 and 12 months after infusion of ZOL in the APR+ group.

The differences in the percent change in total hip BMD after treatment with ZOL suggest that the efficacy of ZOL may be greater in APR+ vs APR− patients. However, the changes in the spine and femoral neck BMD were not significantly different between the APR+ and APR− groups. One of the reasons for this might be the impact of prior bisphosphonate use. However, in this study, only 32 patients (10 in the APR+ group and 22 in the APR− group) were included in the ZOL group, and a washout of at least 2 years was set before administration of ZOL. Thus, the impact of prior bisphosphonate use could be excluded.

We also found that ibuprofen reduced the elevated body temperature as a sign of APRs induced by ZOL infusion. In other studies, acetaminophens were used to ease APR symptoms [18]. Patients in this study could take ibuprofen following development of intolerable fever > 38.5 °C; however, there were no reports of severe APRs, which could be attributed to the successful lowering of fever with the use of ibuprofen in this study. Thus, as in previous reports [17, 18], antipyretic analgesic medication is useful to control the symptoms of APRs in Japanese patients as well.

Some of the limitations of this study include the relatively small sample size in one of the treatment arms, which could have led to the discrepancies in significant factors of APRs vs a previous study [9]. In addition, this study was conducted to confirm the efficacy of ZOL for the treatment of osteoporosis; as such, only limited information could be obtained, and data on the duration of prior bisphosphonate use or which bisphosphonate was being taken were not available for analysis. However, an important strength of the study was that BTM and BMD measurements could be obtained from almost all patients.

## Conclusion

The present analysis showed that APRs appear to occur more frequently in osteoporosis patients with a lower NLR, higher bone turnover, and younger age. Taken together with the results of previous studies, although APRs are frequent in Japanese patients with osteoporosis receiving ZOL, they are manageable with standard treatment such as antipyretic analgesic medications. APRs after the first infusion of ZOL were related to increases in total hip BMD and suppression of BTMs.
